# Expression of HIF-1α/PKM2 axis correlates to biological and clinical significance in papillary thyroid carcinoma

**DOI:** 10.1097/MD.0000000000033232

**Published:** 2023-03-10

**Authors:** Zengfang Hao, Yuan Wang, Jiajun Li, Weina Liu, Wei Zhao, Juan Wang

**Affiliations:** a Department of Pathology, The Second Hospital, Hebei Medical University, Shijiazhuang, China; b Department of Pharmacology, Hebei Medical University, Shijiazhuang, China.

**Keywords:** glycolysis, HIF-1α, papillary thyroid carcinoma, PKM2

## Abstract

hypoxia inducible factor-1α (HIF-1α) and pyruvate kinase M2 (PKM2) are 2 key metabolic regulatory proteins, they could engage in a positive feedback loop and drive cancer growth by enhancing glycolysis. This study aimed to investigate the expression of HIF-1α and PKM2 in papillary thyroid carcinoma (PTC) and its correlation with the patients clinicopathological features and with tumor invasion and metastasis. Surgically resected PTC specimens from 60 patients were collected. The expression levels of HIF-1α and PKM2 in PTC tissues were examined by immunohistochemical staining. The full clinical records of all patients were collected to analyze the relevance between HIF-1α and PKM2 expressions and the clinical pathological features of PTC. The results showed that the positive expressions of HIF-1α, PKM2, and HIF-1α/PKM2 axis (HIF-1α^+^/PKM2^+^) were all significantly higher in PTC than those in normal thyroid follicular epithelium, and a positive correlation was found between HIF-1α and PKM2 in PTC. Further analysis showed that in PTC, the positive expression of HIF-1α and HIF-1α/PKM2 axis (HIF-1α^+^/PKM2^+^) were significantly associated with bigger tumor size, moreover, the positive expressions of HIF-1α, PKM2 and HIF-1α/PKM2 axis (HIF-1α^+^/PKM2^+^) were all correlated with capsular invasion and lymph node metastasis, while they were all not correlated with gender, sex and multicentricity of tumor. This study identified HIF-1a/PKM2 axis as potential molecular marker for predicting the invasion and progression of papillary thyroid carcinoma.

## 1. Introduction

Thyroid cancer is one of the most common endocrine malignancy, and the number of estimated new cases of thyroid cancer was ranked fifth in diverse cancer types in 2020,^[1]^ while it was ranked 6th in 2019.^[2]^ Papillary thyroid carcinoma (PTC) is the most common type of thyroid carcinoma, usually with good prognosis following proper and effective treatment. However, some PTCs are highly invasive, leading to reduced survival and quality of life.^[3]^ Therefore, it is important to explore novel biomarkers associated with PTC metastasis and progression.

Glycolysis, also known as Warburg effect, refers to the transformation of glucose into lactate in cancer cells under the aerobic conditions.^[4]^ Pyruvate kinase M2 (PKM2) is a crucial enzyme to metabolic and glycolytic flux, and its upregulation causes a several-fold increase in glucose consumption and lactate production.^[5]^ In addition to its known role as a metabolic enzyme, PKM2 can act as a signaling modulator in cancer development and progression. High levels of PKM2 are expressed in a variety of human tumors, including breast and colon cancer,^[6,7]^ and are reported to promote the Warburg effect in cancer cells. Previously studies had identified that overexpression of PKM2 provides a selective growth advantage for PTC cells through activation of glycolysis, suggesting that aberrant PKM2 overexpression may serve as a novel biomarker and a potential treatment target for PTC.^[8]^

Hypoxia inducible factor, a transcription factor, is a heterodimer of the hypoxia inducible factor-1α (HIF-1α) and HIF-1β subunits and has a key role in tumor cells for energy production for maintaining their metabolism. Normally, HIF-1α undergoes quick, proteasome-mediated degradation, but under hypoxic conditions, it is stabilized. It activates the transcription of genes that are involved in crucial aspects of cancer biology, including angiogenesis, cell survival, glucose metabolism and invasion. Various cancers include liver, breast, cervix, and oropharynx all overexpressed HIF-1α and its overexpression often positively leads to poor prognosis.^[9]^ Liu et al^[10]^ found that significantly higher positive rates (73%) of HIF-1α were observed in PTC compared with the control group (27%) by immunohistochemistry (IHC).

PKM2 was recently proposed to be part of a “feed forward loop” enhancing the activity of HIF-1α. Correlation between PKM2 and HIF-1α is complicated. HIF-1α regulates PKM2 expression by binding to the hypoxia-response elements located within the first intron of the PKM2 gene, thus regulating cell metabolism. Additionally, PKM2 increases the binding of HIF-1α and the recruitment of the coactivator p300 to hypoxia-response elements on HIF-1α target genes in a positive feedback loop.^[11]^ A large body of data indicates that HIF-1α/PKM2 loop contribute to the Warburg effect and tumor growth.^[12,13]^ Hua et al^[14]^ reported that the positive feedback loop of PKM2/HIFα axis paves the way to cancer-specific metabolism in response to low oxygen-glucose challenge in non-small cell lung cancer (NSCLC), and targeting this axis may present new perspectives in the prevention or treatment of NSCLC. However, research about the relationship between HIF-1α/PKM2 axis and the progression of papillary thyroid carcinoma is still scare.

Thus, the aim of this study was to investigate the expression of HIF-1α/PKM2 axis in papillary thyroid carcinoma and its roles in the development of PTC.

## 2. Material and Methods

### 2.1. Study participants

Formalin-fixed and paraffin-embedded resection tumor tissues and paired normal thyroid tissues of 60 PTC patients from the archives of the Department of Pathology, the Second Hospital of Hebei Medical University, were obtained and subjected to IHC analyses.

#### 2.1.1. The inclusion criteria were.

(1) Pathologically confirmed PTC.
(2) Initial curative surgical resection.

#### 2.2.1. Exclusion criteria of participants.

(1) With malignancy except PTC.
(2) The patients had undergone other chemo- or radio- therapies before surgery.

The morphologic classification of the adenocarcinomas was conducted according to the 4th edition of World Health Organization criteria, whereas the staging was based on the 8th edition of TNM system. This investigation was approved by the Ethics Committee of the Second Hospital of Hebei Medical University.

### 2.2. IHC

Sections (4 μm thick) were prepared from paraffin blocks. After deparaffinization, antigen retrieval was performed under citrate buffer for 5 minutes. Endogenous peroxidase activity was blocked with 3% hydrogen peroxide in methanol for 30 minutes. Incubation with primary antibodies against HIF-1α (1:200, Genetex, Inc, Irvine, CA) and PKM2 (1:400, Cell Signaling Technology Inc, Danvers, MA), was conducted overnight at 4°C in a humidified chamber. After washing with phosphate-buffered saline, the sections were stained according to the instructions of SP kit (ZSGB- Bio). Counerstaining was performed with hematoxylin. Paralleled staining was performed in the absence of the primary antibody to serve as negative controls.

### 2.3. Immunohistochemical staining evaluation

To rule out the possibility of an interpersonal bias, the results were interpreted by 2 pathologists who were blind to the clinical outcome. Brown granules in cytoplasm or nucleus represented positive IHC results. Then the positive cell percentage and the positive expression intensity were recorded. Ten high magnification fields (magnification: 400 ×) were randomly selected for observation. The number of positively stained cells were quantified as a percentage of total number of tumor cells counted. The scoring criteria of the positive cell percentage: 0: Positive cell ≤ 5%; Positive cell 6% to 25%; Positive cell 26% to 50%; Positive cell 51% to 75%; Positive cell ≥ 76%. The scoring criteria of the positive expression intensity: 0: No colorization; Yellow-colored; Yellow brown-colored; Dark brown-colored. The final result of HIF-1α and PKM2 immunohistochemical test was acquired from the product of positive cell score and positive intensity score. The cutoff point was the median (score < 8, negative expression and ≥ 8, positive expression).^[15,16]^

### 2.4. Statistical analysis

The experimental data were statistically analyzed with Chi-squared test, Fisher exact test, and correlation using the SPSS software 16.0 (SPSS Inc., Chicago, IL). *P* < .05 was considered statistically significant.

## 3. Results

### 3.1. Patient characters

The mean age of PTC patients was 43.4 ± 13.2 years, and there were 43 (71.7%) females/17 (28.3%) males (Table 1). Regarding tumor features, the mean tumor size was 2.0 ± 1.5 cm; 41 (68.3%) patients had capsule invasion; 24 (60.0%) patients presented multicentricity. Thirty-four (56.7%) patients exhibited lymph node metastases.

**Table 1:** The clinicopathological features of 60 PTC patients.

Clinical characteristics	Overall (n)
All	60
Age	
<55 yr	47
≥55 yr	13
Sex	
Male	17
Female	43
Tumor size	
≤1cm	14
>1cm	46
Multicentricity	
Absent	36
Present	24
Capsular invasion	
Absent	19
Present	41
Lymph node metastasis	
Absent	26
Present	34

### 3.2. The expressions of HIF-1α/PKM2 axis proteins in PTC tissues were different from normal thyroid follicular epithelium

**Figure 1.**
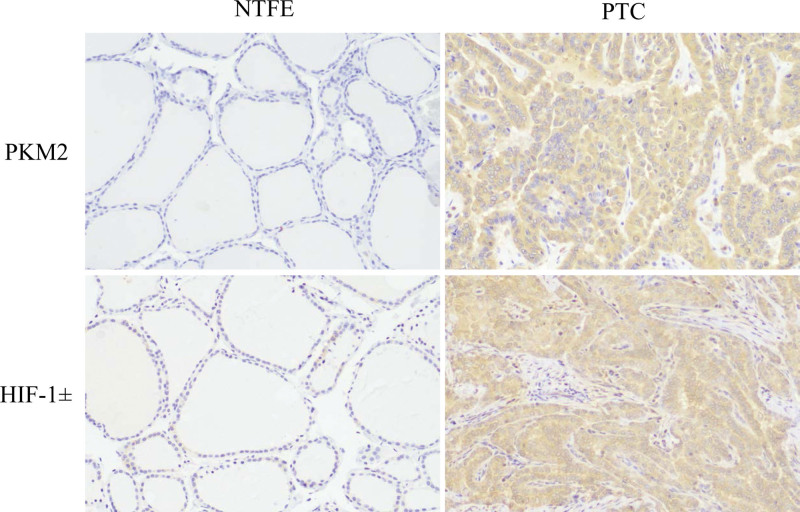
Different expressions of HIF-1α and PKM2 in normal thyroid follicular epithelium (NTFE) and papillary thyroid carcinoma (PTC). HIF-1α = hypoxia inducible factor-1α, PKM2 = pyruvate kinase M2.

**Figure 2.**
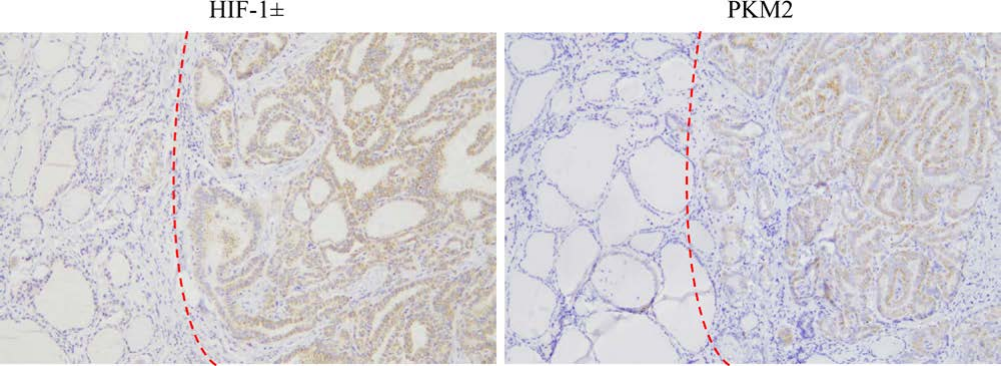
The expressions of HIF-1α and PKM2 were positive in PTC (the right half of the picture) but negative in the follicular epithelium adjacent to the PTC (the left half of the picture). HIF-1α = hypoxia inducible factor-1α, PKM2 = pyruvate kinase M2, PTC = papillary thyroid carcinoma.

Immunohistochemical results showed that the percentage of HIF-1α-positive expression was 78.3% among 60 cases with PTC and was significantly higher than that of normal thyroid follicular epithelium (35.0%, *P* < .05). PKM2-positive expression occurred more frequently in all cases of PTC than that in normal thyroid follicular epithelium (75.0 % vs 28.3%, *P* < .05). In addition, the immunophenotyping abnormality (HIF-1α^+^/PKM2^+^) of HIF-1α/PKM2 axis was found in 65.0% of PTC cases, whereas a lower abnormality was seen in normal thyroid follicular epithelium (10.0%; Table 2, Figs. 1 and 2). Moreover, spearman correlation analysis indicated that HIF-1α IHC scores were positively correlated with PKM2 IHC scores in PTC tissues (*P* < .05, *R* = 0.307) (Table 3).

**Table 2:** Expressions of HIF-1α and PKM2 in normal thyroid follicular epithelium and papillary thyroid carcinoma.

Groups	n	n(%)		
		HIF-1α^+^	PKM2^+^	HIF-1α^+^/PKM2^+^
NTFE	60	21 (35.0)	17 (28.3)	6 (10.0)
PTC	60	47 (78.3)*	45 (75.0)*	39 (65.0)*

HIF-1α = hypoxia inducible factor-1α, NTFE = normal thyroid follicular epithelium, PKM2 = Pyruvate kinase M2, PTC = papillary thyroid carcinoma.

* *P* < .05 (PTC vs NTFE).

**Table 3:** Relationship between the expression of HIF-1α and PKM2 in PTC.

		HIF-1α	
		−	+
PKM2	−	7	8
	+	6	39
*P* < .05, *R* = 0.350			

HIF-1α = hypoxia inducible factor-1α, PKM2 = pyruvate kinase M2, PTC = papillary thyroid carcinoma.

The above results indicated that the positive expressions of HIF-1α, PKM2 and HIF-1α/PKM2 axis (HIF-1α^+^/PKM2^+^) were all significantly higher in PTC than those in normal thyroid follicular epithelium, and a positive correlation was found between HIF-1α and PKM2 in PTC.

### 3.3. The clinicopathologic significance of HIF-1α/PKM2 feedback loop proteins in PTC

We next explored the correlation between HIF-1α/PKM2 feedback loop proteins expression and clinicopathologic parameters in Table 4, the percentage of positive expression of HIF-1α was closely related with tumor size, being significantly higher in PTC with bigger tumor size than that in cases with smaller tumor size (87.0% vs 50.0%, *P* < .05). Both the positive expressions of HIF-1α and PKM2 were observed more frequently in cases with capsular invasion than those of without (90.2% vs 52.6%, 85.4% vs 52.6%, both *P* < .05) and in cases with lymph node metastasis than those without lymph node metastasis (88.2% vs 65.4%, 88.2% vs 57.7% both *P* < .05).

**Table 4:** Relationships of HIF-1α/PKM2 feedback loop proteins with clinical pathological characteristics in PTC.

Pathological characteristics	HIF-1α^+^	PKM2^+^	HIF-1α^+^/PKM2^+^
Age			
<55 years	36 (76.6)	35 (74.5)	31 (66.0)
≥55 years	11 (84.6)	10 (76.9)	8 (61.5)
Sex			
Male	14 (82.4)	11 (64.7)	10 (58.8)
Female	33 (76.7)	34 (79.1)	29 (67.4)
Tumor size			
≤1cm	7 (50.0)	8 (57.1)	4 (28.6)
>1cm	40 (87.0)*	37 (80.4)	35 (76.1)*
Multicentricity			
Absent	27 (75.0)	26 (72.2)	21 (58.3)
Present	20 (83.3)	19 (79.1)	18 (75.0)
Capsular invasion			
Absent	10 (52.6)	10 (52.6)	7 (36.8)
Present	37 (90.2)†	35 (85.4)†	32 (78.0)†
Lymph node metastasis			
Absent	17 (65.4)	15 (57.7)	11 (42.3)
Present	30 (88.2)‡	30 (88.2)‡	28 (82.4)‡

HIF-1α = hypoxia inducible factor-1α, PKM2 = pyruvate kinase M2, PTC = papillary thyroid carcinoma.

* *P* < .05 (tumor size of > 1cm vs ≤ 1cm in PTC).

† *P* < .05 (with capsular invasion vs without capsular invasion in PTC).

‡ *P* < .05 (lymph node metastasis vs without lymph node metastasis in PTC).

Based on the above findings, we also summarized the association of HIF-1α/PKM2 feedback loop phenotype abnormalities and clinicopathologic significance in PTC. Phenotype abnormality of this axis (HIF-1α^+^/PKM2^+^) was significantly correlated with bigger tumor size (76.1% vs 28.6%), capsular invasion (78.0% vs 36.8%) and lymph node metastasis (82.4% vs 42.3%) (*P* < .05) (Table 4).

The above results demonstrated that the expressions of HIF-1α and HIF-1α/PKM2 axis (HIF-1α^+^/PKM2^+^) were significantly associated with bigger tumor size, and the expressions of HIF-1α, PKM2 and HIF-1α/PKM2 axis were all correlated with capsular invasion and lymph node metastasis.

## 4. Discussion

HIF-1α and PKM2 engage in a feedback loop, enhancing activity of both key metabolic regulatory proteins, and it is an important mechanism in the development and progression of many human malignancies.^[17–20]^ However, the role of this feedback loop in PTC as well as their correlation with clinical characteristics is still not clarified. In the present study, the expression and clinicopathologic significance of the HIF-1α and PKM2 protein between PTC and normal thyroid follicular epithelium were comparatively investigated. Our results showed that the abnormalities of HIF-1α/PKM2 loop, including alterations in single and combined members, were all significantly higher in PTC than those in normal thyroid follicular epithelium. Moreover, the expression pattern of the 2 proteins of this loop indicated a different relationship with clinicopathologic parameters in PTC. To our knowledge, ours is the first preliminary results suggesting that different expression pattern and clinicopathologic significance of the HIF-1α/PKM2 loop do exist between PTC and normal thyroid follicular epithelium, indicating that the expression of HIF-1α/PKM2 loop may impact PTC cells of growth.

About 80 years ago, Otto Warburg found that most cancer cells relied primarily on aerobic glycolysis to generate energy even in an oxygen-rich environment, which is termed “the Warburg effect.” As the last rate limiting enzyme in glycolysis, PKM2 plays a crucial part in regulating cancer cells metabolism and tumorigenesis. The PKM2 mRNA levels have been reported to be elevated in breast cancer, colon cancer, and bladder cancer.^[21–24]^ IHC results further showed that PKM2 was overexpressed in many malignant tumors and made essential contributions in development and metastasis.^[25,26]^ PKM2 expression has been associated with esophageal squamous cell carcinoma chemoresistance,^[18]^ while its knockdown in NSCLC increased the radiosensitivity of resistant cell lines.^[19]^ In this study, we found that PKM2 was highly expressed in PTC tissues and related to the capsular invasion and lymph node metastasis of PTC patients, which was in line with a previous report.^[8]^ Though not statistically significant, we also found an increased trend of PKM2 in tumors with bigger tumor size, which may predict a poorer clinical outcome in PTC patients.

PKM2 was recently proposed to be part of a “feed forward loop” enhancing the activity of HIF-1α, a key transcriptional regulator of both aerobic and anaerobic glycolysis. Indeed, HIF-1α can activate pkm2 gene transcription, and PKM2 in turn interacts with the HIF-1a subunit and promotes trans-activation of hypoxia inducible factor target genes, thus enhancing cellular responses to oxygen deprivation or oncogene activation.^[9]^ Dysregulation of HIF is increasingly recognized as a critical step during cancer progression. Deletion of HIF-1α has been reported to markedly impair metastasis in a mouse mammary tumor virus promoter-driven polyoma middle T antigen mouse model of breast cancer.^[27]^ In an orthotopic xenograft model of lung cancer, the HIF-1α antagonist PX-478 effectively inhibits tumor progression.^[28]^ Similarly, we identified a high HIF-1α protein expression in PTC tissues, the expression of HIF-1α was also related to tumor size, capsular invasion and lymph node metastasis, revealing an important role of HIF-1α in pathogenesis of PTC. Furthermore, Spearman correlation analysis of the IHC results showed a significant positive correlation between PKM2 and HIF-1α, indicating that PKM2/HIF-1α loop may contribute to the development and progression of PTC.

In conclusion, these data demonstrated that different expression and clinicopathologic significance of the HIF-1α/PKM2 feedback loop did exist between PTC and normal thyroid tissues, supporting the linkage between HIF-1α/PKM2 feedback loop and the progression of papillary thyroid carcinoma.

## Author contributions

**Conceptualization:** Juan Wang.

**Data curation:** Zengfang Hao, Yuan Wang.

**Funding acquisition:** Zengfang Hao, Yuan Wang.

**Investigation:** Zengfang Hao, Yuan Wang, Jiajun Li, Weina Liu, Wei Zhao.

**Methodology:** Zengfang Hao, Yuan Wang, Jiajun Li, Weina Liu, Wei Zhao.

**Supervision:** Juan Wang.

**Writing – original draft:** Zengfang Hao, Yuan Wang.

**Writing – review & editing:** Juan Wang.
